# A Wireless Passive Pressure Microsensor Fabricated in HTCC MEMS Technology for Harsh Environments

**DOI:** 10.3390/s130809896

**Published:** 2013-08-02

**Authors:** Qiulin Tan, Hao Kang, Jijun Xiong, Li Qin, Wendong Zhang, Chen Li, Liqiong Ding, Xiansheng Zhang, Mingliang Yang

**Affiliations:** 1 Key Laboratory of Instrumentation Science & Dynamic Measurement, Ministry of Education, North University of China, Taiyuan 030051, China; E-Mails: tanqiulin@nuc.edu.cn (Q.T.); 18734135497@163.com (H.K); qinli@nuc.edu.cn (L.Q.); wdzhang@nuc.edu.cn (W.Z.); lchen417@163.com (C.L.); dingliqiong5@163.com (L.D.); xiansheng8709@163.com (X.Z.); yangmingliang418@163.com (M.Y.); 2 Science and Technology on Electronic Test & Measurement Laboratory, North University of China, Taiyuan 030051, China

**Keywords:** wireless passive, high-temperature co-fired ceramics (HTCC), LC circuit mutual inductance coupling

## Abstract

A wireless passive high-temperature pressure sensor without evacuation channel fabricated in high-temperature co-fired ceramics (HTCC) technology is proposed. The properties of the HTCC material ensure the sensor can be applied in harsh environments. The sensor without evacuation channel can be completely gastight. The wireless data is obtained with a reader antenna by mutual inductance coupling. Experimental systems are designed to obtain the frequency-pressure characteristic, frequency-temperature characteristic and coupling distance. Experimental results show that the sensor can be coupled with an antenna at 600 °C and max distance of 2.8 cm at room temperature. The senor sensitivity is about 860 Hz/bar and hysteresis error and repeatability error are quite low.

## 1. Introduction

Nowadays, high temperature pressure sensors has been attached great importance due to their excellent work capability in harsh temperature environments such as in automobiles [1], aero engine turbines [2], aeronautics [3] and is one of the important areas in the sensor research [4]. High temperature pressure sensors have broad application prospects in the area of civil industry and national defense [5].

With the fast development in the last decades of micromachining technology, micro-electromechanical system (MEMS) sensors are the major devices used in measuring pressure [6]. Most pressure sensors are made from silicon. However, silicon sensors with PN-junctions exhibit a drawback, which is they cannot be used above 150 °C, since the leakage current across the junctions drastically increases at 150 °C [7]. Furthermore, above 500 °C, the mechanical properties of silicon will deteriorate as the material becomes easily deformable when pressure is applied [8]. Using SOI material can increase the operation temperature of sensor, but sensor become invalid because the silicon material will lose elasticity at 500 °C [9,10]. The Georgia Institute of Technology has designed a wireless high temperature pressure sensor using low temperature co-fired ceramic (LTCC) material. However, the sensor is only tested to 450 °C [11–14]. Another team in Novi Sad (Serbia) demonstrated in 2009 a better structure, but worse performance [15,16]. Two LTCC layers are added above the metal electrode to provide perfect protection, but the sensor sensitivity is only 25.6 kHz/bar. Xiong, *et al.* [17] have showed the measurement and fabrication of wireless pressure sensor fabricated in HTCC MEMS technology, but the sensor has not been tested in high temperature environments. Recently Jie Yang has introduced a harsh environment wireless pressure sensing solution utilizing high temperature electronics; the proposed approach was verified through prototype fabrication and high temperature bench testing from room temperature up to 450 °C [18].

A pressure sensor based on HTCC technology is proposed. The non-contact measurement technology and HTCC material are involved to solve the problem of lead degradation and elastic properties deterioration of materials at ultra-high temperature, respectively [19].The sensor in this article does not have evacuation channel and exit hole in order to ensure the sensor is completely gastight in harsh environments. The wireless resonant sensor is based on an LC circuit. By using this signal extraction method, the sensor has great potential for enlarging the working temperature range and a great advantage in miniaturization and its self-adaptive package [20].

A precision frequency-pressure characteristic measurement system is built to measure the sensor resonant frequency at room temperature. The sensor resonant frequency is read by extracting the impedance phase dependences of the antenna coil. Furthermore, the sensor frequency-temperature characteristic and coupling distance have been measured with high temperature measurement system and coupling distance testing platform, respectively.

## 2. Sensor Design

The sensor designed in this paper can be equal to a series LC resonant circuit which consists of variable capacitance and invariable inductance, and is powered by a reader antenna [21]. The resonant frequency can be retrieved from the expression:

(1) $${\text{f}}_{0}=\frac{1}{2\pi \sqrt{L_{s}C_{s}}}$$

Where *L_s_* and *C_s_* are sensor inductance and capacitance respectively. When pressure is applied to the membrane, the cavity gap becomes narrow, and then the capacitance increases [22].

**Figure 1.**
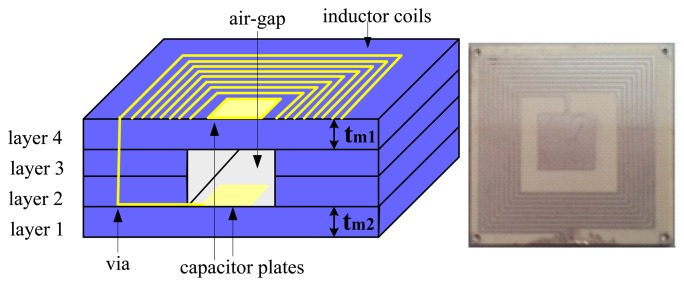
Cross section of sensor and fabricated sensor.

The cross section of sensor model which consists of four green tapes is shown in Figure 1. The inductor is designed as a square spiral type and placed on the layer 4. Relevant geometrical parameters of the inductor are given in Table 1. The capacitor is a parallel plate type with square electrodes. The lower capacitor electrode is placed on the top side of the layer 1, while the upper electrode resides on the top side of the layer 4. The relevant geometrical parameters of the capacitor are given in Table 2. An air-gap is present between the two capacitor electrodes. Electrical connection between capacitor plates and inductor is realized with metalized via.

Tape layers with the capacitor electrodes act as sensor membranes which react to pressure. The sensor presented in this paper does not have an evacuation channel in order to ensure the sensor is completely gastight. The carbon membrane which prevents collapse of cavity during lamination will become carbon dioxide during co-firing and spread to air through the small pores which are formed after volatilization of organics. Characteristics of the implemented tape are shown in Table 3.

## 3. Model Analysis

**Figure 2.**
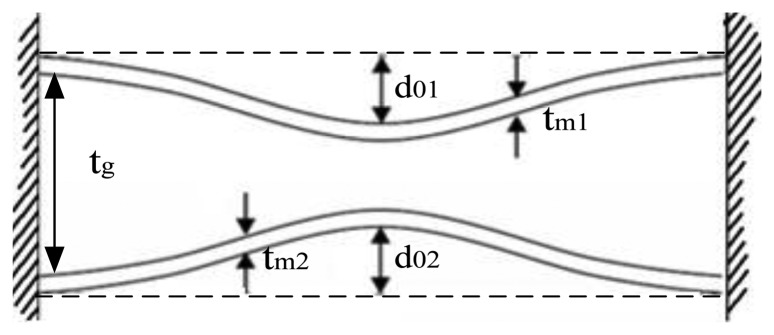
Deflection of membranes as a result of applied pressure.

The variations of pressure signal are detected by changes in the sensors' capacitance as shown in Figure 2. When the sensor has been fabricated, the pressure in cavity is about 0.166 atm, which is smaller than the outside pressure of 1 atm. It means that the pressure of 0.834 atm is initially applied on the membrane, so the initial square parallel capacitance can be determined as [23]:

(2) $$C_{0}=\frac{\frac{{ɛ}_{0}a_{e}^{2}}{t_{g}+\frac{t_{m1}}{{ɛ}_{r}}}}{\sqrt{\frac{d_{01}(0.834\text{atm})+d_{02}(0.834\text{atm})}{t_{g}+\frac{t_{m1}}{{ɛ}_{r}}}}}\tanh^{-1}\left(\sqrt{\frac{d_{01}(0.834\text{atm})+d_{02}(0.834\text{atm})}{t_{g}+\frac{t_{m1}}{{ɛ}_{r}}}}\right)$$

where ε_r_ and ε_0_ are the permittivity of the tape material and the dielectric constant of air, respectively, t_g_ denotes cavity thickness, a_e_ represents length of one side of electrode, t_m1_ stands for upper diaphragm thickness, d_01_(0.834 atm) and d_02_(0.834 atm) are the center deflection of the upper and lower membranes under 0.834 atm, respectively.

**Figure 3.**
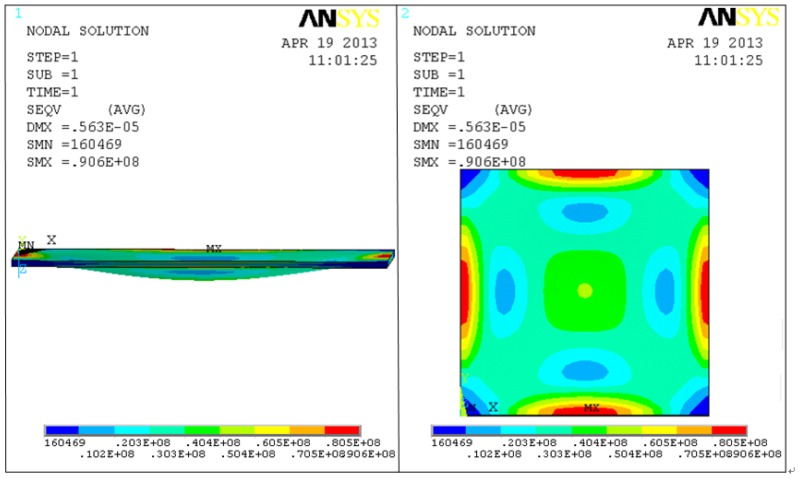
ANSYS simulation of membrane deflection under 0.1 MPa.

The membrane is deformed when pressure is applied, which is shown schematically in Figure 3. When pressure is applied on membranes, the sensor capacitance can be derived using the expression:

(3) $$C_{\text{plate}}(P)=\frac{C_{0}}{\sqrt{\frac{d_{01}+d_{02}}{t_{g}+\frac{t_{m1}}{{ɛ}_{r}}}}}\tanh^{-1}\left(\sqrt{\frac{d_{01}+d_{02}}{t_{g}+\frac{t_{m1}}{{ɛ}_{r}}}}\right)$$

where d_01_ and d_02_ given in [24] denote the center deflection of the upper and lower membranes under applied pressure respectively. d_01_ and d_02_ under 0.1 MPa are equal for the sensor in this paper and calculated value of d_01_ and d_02_ almost fit the result of ANSYS simulation shown in Figure 3.

**Figure 4.**
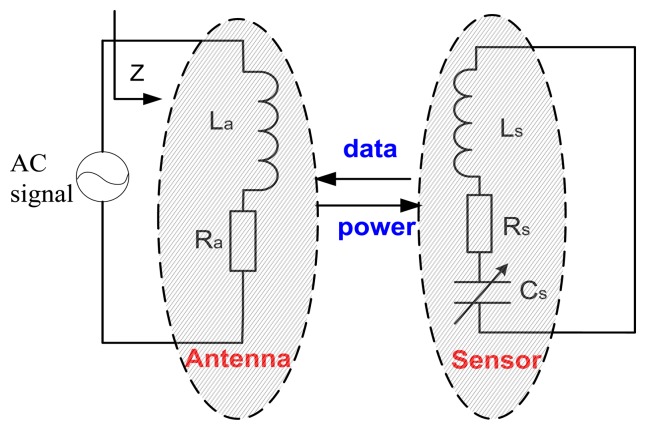
Model of the sensor-antenna system.

A wireless measurement technique is provided for data retrieval [25]. The electrical model of the sensor-antenna system is presented in Figure 4 [26].

The alternating signal which is input through the sweeping frequency at the signal test end is sent out to the inductance coil of sensor in the form of electromagnetic energy by the reader antenna coil. When the signal frequency is equal to the sensor resonance frequency, the input impedance of antenna will change apparently [27]. The sensor resonant frequency can be obtained through testing antenna impedance phase with Agilent the E4991A Impedance Analyzer. The total impedance of the reader antenna can be determined as:

(4) $$Z=R_{a}+j2\pi fL_{a}\left[1+\frac{k^{2}{\left(\frac{f}{f_{0}}\right)}^{2}}{1+j\frac{1}{Q}\frac{f}{f_{0}}-{\left(\frac{f}{f_{0}}\right)}^{2}}\right]$$

where R_a_ is series resistance of reader antenna, L_a_ is inductance of reader antenna, Q is the quality factor of sensor. From Equation (4), it is obvious that Z relates to the sensor resonance frequency f_0_, and f_0_ can be obtained by measuring input impedance Z.

## 4. Fabrication

**Figure 5.**
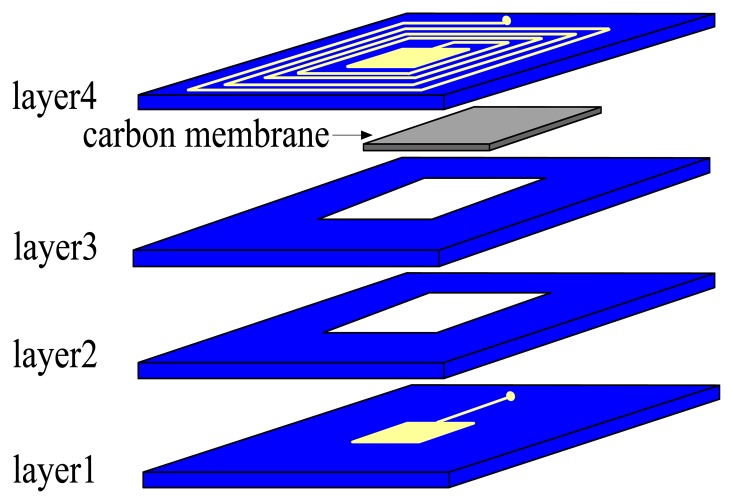
Part of fabrication processes: Stack cutted and metallized layers include layer 1, layer 2 and layer 3 successively and place carbon membrane in the cavity. Then stack layer 4 on the layer 3.

The sensor is fabricated in HTCC technology which covers more than 10 processes include milling, tape casting, cutting, hole drilling, screen-printing, stacking, isostatic lamination, firing, *etc.* [28]. Part of THE fabrication process is shown in Figure 5.

The first step is to cut THE via and cavity using a drill. Then the screen-printing technique is applied for printing of metal figure and via filling. Platinum paste is used for metallization printing and via filling and its properties are shown in Table 4. Afterwards, the printed tapes are put in a furnace for 20 min at 100 °C. The next process is to stack Section 1, include layer 1, layer 2 and layer 3 and placing of carbon membrane in the cavity. After that, stack layer 4 is applied on Section 1.

Isostatic lamination of the HTCC layers has been performed at a pressure of 15 MPa and temperature of 75 °C for 20 min after vacuum packaging. The laminated stack is sintered in a furnace and heated 430 °C (1 °C/min ramp rate) in air and heated 600 °C (2 °C/min ramp rate) and then for 126 min at a peak temperature of 1,512 °C (12.5 °C/min ramp rate). The geometrical parameters of the sensor design after fabrication is shown in Table 5.

**Figure 6.**
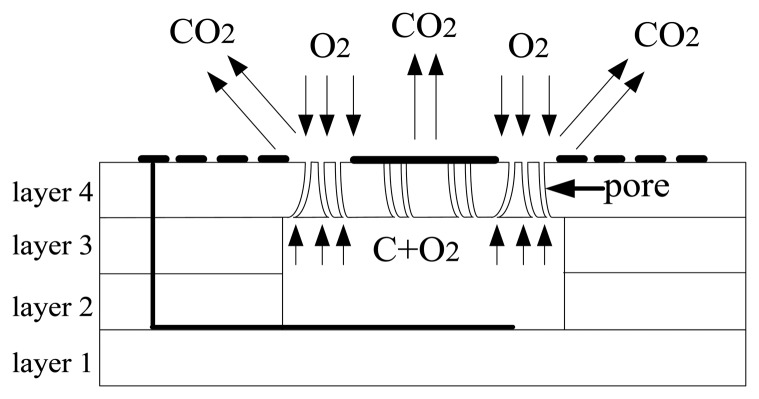
Formation of cavity: The carbon membrane turns into CO_2_ and spreads to air through the pores

**Figure 7.**
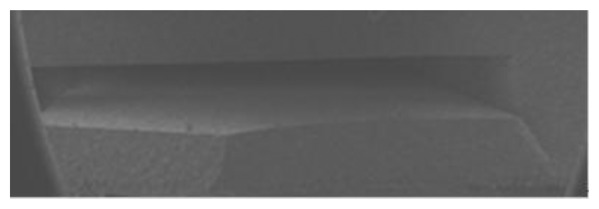
SEM analysis of cavity.

The cavity formation is shown in Figure 6. Organics in the substrate decompose severely at 100–360 °C in the sintering process; the decomposition of organics in the substrate includes two processes [29]: removal of volatile organic and volatile product. The first stage refers to the volatilization of organic solvents and the removal of plasticizer and other small molecule organics. The organics can spread to the surface of the HTCC tape, and then diffuse to air due to the sufficient vapor pressure in HTCC tape. The second stage is that volatile decomposition products are produced constantly during the process of thermal decomposition and spread to the surface to air. Then the pores are formed after decomposition of organics in substrate. The carbon membrane turns into CO_2_ at about 600 °C and spreads to air through the pores. CO_2_ can also spread to air thru the pores which is covered by the electrode because the ceramic tape and platinum paste are not impermeable at 600 °C. The green tape will be impermeable after a full sintering process. SEM analysis of cavity is presented in Figure 7. As can be seen in Figure 7, the cavity is preserved well.

## 5. Experiments

**Figure 8.**
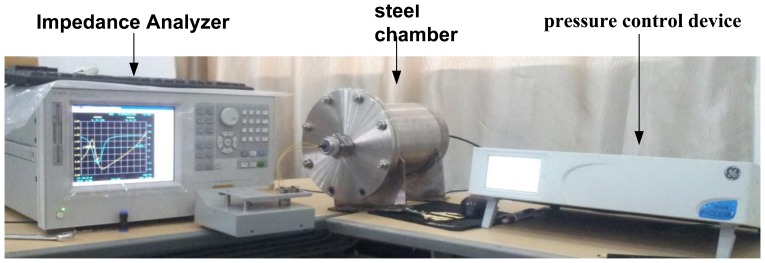
Frequency-pressure measurement system.

A frequency-pressure measurement system which consists of an Agilent E4991A Impedance Analyzer, steel chamber, pressure control device and nitrogen pressure tank as shown in Figure 8 is designed in order to measure the sensor resonant frequency at different pressures at room temperature. The antenna and sensor are a certain distance apart without any electric connection inside the chamber.

**Figure 9.**
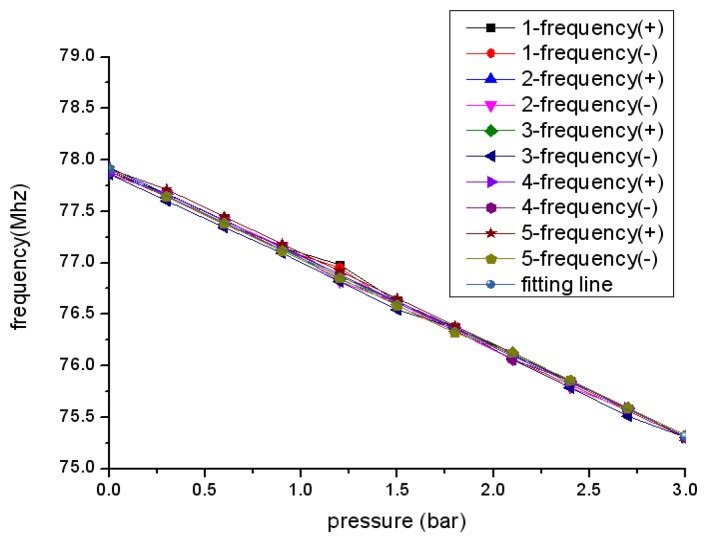
Measured resonant frequency *versus* pressure characteristic.

The sensor output signal has been measured in the form of positive route and reverse route five times respectively. The measured sensor characteristic is shown in Figure 9. As can be seen, the change of sensor resonant frequency dependence on pressure approximates a linear variation.

The linearity, hysteresis error, repeatability error of sensor can be determined as:

(5) $$\delta_{\text{L},\text{H},\text{R}}=\frac{\Delta_{\text{max}}}{{\text{y}}_{\text{F}*\text{S}}}\times 100\%$$

The linearity δ_L_, hysteresis error δ_H_ and repeatability error δ_R_ are 96.56%, 3.12% and 6.03%, respectively. The sensor resonant frequency at zero pressure is 77.76 MHz, which is larger than the calculated value. This is expected because the dimensions of metallization and green tape become small after sintering. Specifically, the inductance becomes small because the dimensions of inductance become small. Both of capacitor electrodes area and distance between electrodes become small, however decrease of capacitor electrodes area makes more contribution, finally the capacitor becomes small. The frequency calculated using dimensions after fabrication is still smaller than the measured value and the reason can be obtained from the Equation (6):

(6) $$f=f_{0}\left(1+\frac{k^{2}}{4}+\frac{1}{8Q^{2}}\right)$$

**Figure 10.**
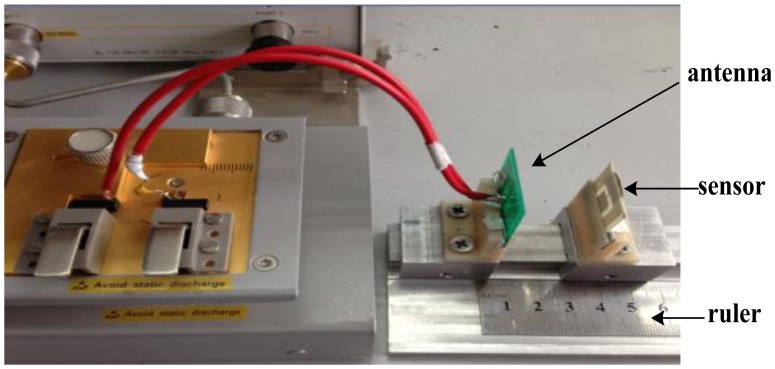
Coupling distance testing platform.

Where f_0_ is theoretical value, f is the measured value, k denotes the coupling coefficient and Q is the quality factor. It is clear that the measured value relates to the coupling coefficient and quality factor and the measured frequency f is greater than the theoretical value. The sensitivity of the sensor is 860 KHz/bar which is lower than the calculated sensitivity since the dimensions of the ceramic tape and metal figure include inductance and capacitor change after fabrication and membrane is initially deformed caused by the fabrication process and pressure difference between inside and out. The presented sensor sensitivity is higher compared to the ones presented in [30,31]. In addition, the measurement result may be influenced by the measurement setup and surrounding environment. In addition, the coupling distance which can be tested by the platform shown in Figure 10 can be 2.8 cm.

**Figure 11.**
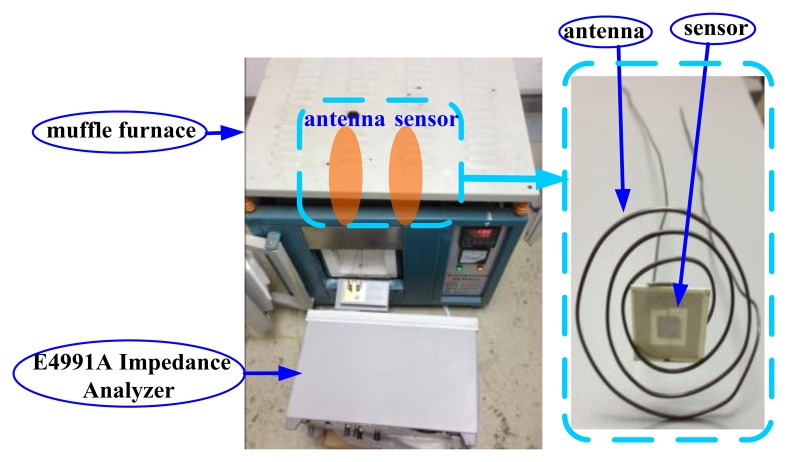
High temperature measurement system.

The sensor has been tested as a function of temperature from 25 °C to 600 °C with the high temperature measurement system consisting of a muffle furnace and impedance analyzer shown in Figure 11. The antenna measured at high temperature is coiled tungsten wire due to its better stability at high temperature. The antenna and sensor are measured in the muffle furnace and impedance analyzer is in the room temperature environment.

**Figure 12.**
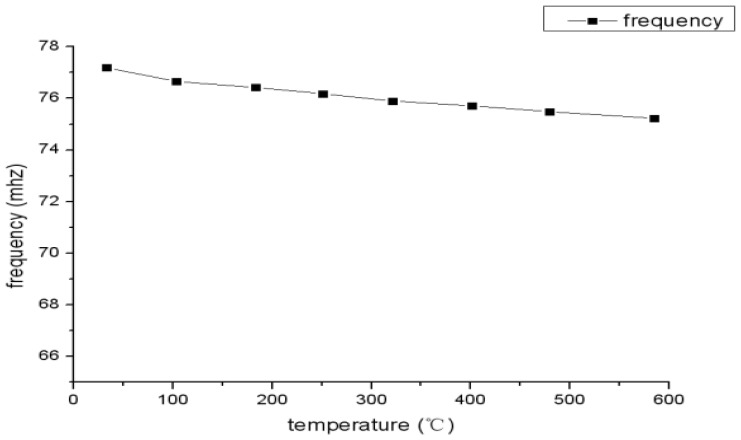
Sensor resonant frequency *versus* temperature.

**Figure 13.**
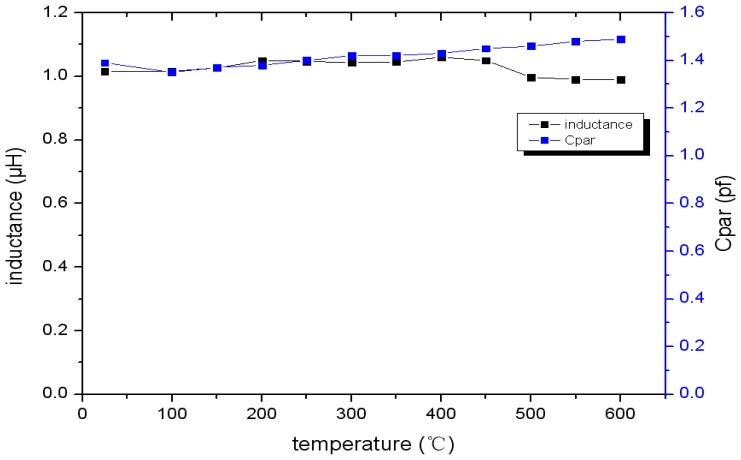
Inductance and parasitic capacitance of the inductance coil *versus* temperature.

**Figure 14.**
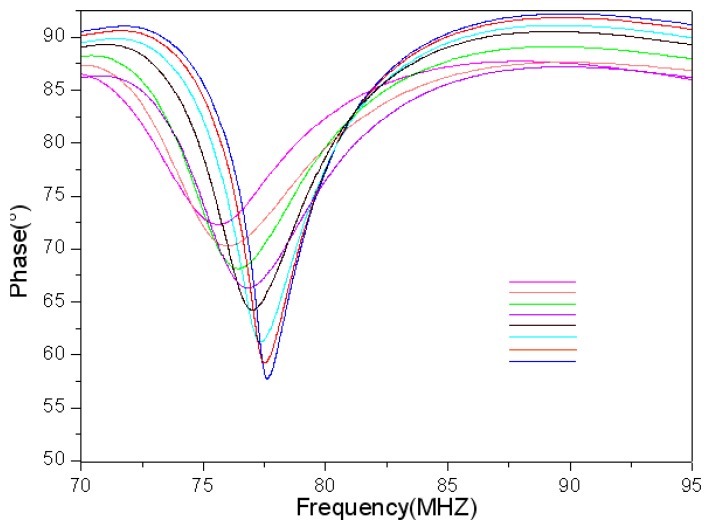
Measured impedance phase *versus* sensor frequency from 25 °C to 600 °C.

From the measurement results shown in Figure 12 and Figure 13, it is clear that the frequency reduces as the temperature increases and the inductance and parasitic capacitance of the inductance coil change slightly. Therefore, the inductance and parasitic capacitance of the inductance coil have little effect on the decrease of sensor resonance frequency as the temperature increases. Further, the sensor can be coupled with the tungsten antenna at 600 °C, but the coupling effect at 600 °C is impaired apparently compared with at 25 °C, as shown in Figure 14. In order to strengthen the coupling effect and measure the sensor at higher temperatures, the geometry parameter of inductance coil should be optimized.

## 6. Conclusions

The presented resonant pressure sensor applying standard HTCC technology is realized with a passive LC circuit. The proposed sensor is a good choice for applications in harsh environments because of the excellent properties of the HTCC material in such environments. The sensor presented does not have an evacuation channel, therefore, there is no need to seal the evacuation channel and the sensor is completely gastight in harsh environments. Pressure variations are detected by a change in sensor resonant frequency. The experimental results show that the sensor data can be detected at 600 °C and a maximum distance of 2.8 cm at room temperature. The sensor exhibits an almost linear change of the resonant frequency and a sizable sensitivity. Besides, the nonlinearity, hysteresis error and repeatability error are quite low.

Future work will be directed towards improving the sensitivity of the sensor and enlarge the sensor measurement temperature range by optimizing the geometric parameters of the inductance coil and measuring the temperature characteristics of the electrode capacitance of sensor. Further, by using the green tape with lower Young's modulus and enlarging the electrode area the sensor sensitivity can also be improved.

## Figures and Tables

**Table 1.** Geometrical parameters of inductor design.

**Symbol**	**Quantity**	**Value**
din	inner diameter of the inductor coil	12 mm
lw	width of inductor coil	300 μm
ls	spacing between adjacent segments	300 μm
n	number of inductor coil	8

**Table 2.** Geometrical parameters of capacitor design.

**Symbol**	**Quantity**	**Value**
a	length of a side of cavity	7 mm
a_e_	length of a side of electrode	6.2 mm
t_g_	cavity thickness	200 μm

**Table 3.** Characteristics of HTCC tape.

**Quantity**	**Value**
Young's modulus	380 GPa
Poisson's Ratio	0.24
CTE	7.7 ppm·C^−1^
Unfired thickness	100 μm
X,Y,Z Shrinkage	14 ± 1%
relative permittivity	9

**Table 4.** Properties of platinum paste.

**Property**	**Value**
TCR	0.00374/°C
Sheet resistance	30 ± 10 mΩ/sq
Firing temperature	1,500 ± 15 °C

**Table 5.** The geometrical parameters of the sensor design after fabrication

**Quantity**	**Value**
Inner diameter of the inductor coil	10.7 mm
Length of a side of electrode	5.5 mm
Sensor dimension	24.5 × 24.5 mm
